# Prevalence and Risk Factors of Hypertension Among Adults in Ernakulam District, Kerala: A Cross-Sectional Study

**DOI:** 10.7759/cureus.81117

**Published:** 2025-03-24

**Authors:** Aparna Ajay, Paul T Francis, Aswathy Sreedevi, Rajeesh R Nair, Lalithambika CV

**Affiliations:** 1 Community Medicine, Amrita Institute of Medical Sciences and Research Center, Kochi, IND; 2 Physiology, Amrita Institute of Medical Sciences and Research Center, Kochi, IND

**Keywords:** blood pressure, hypertension, psqi, risk factors, sleep quality

## Abstract

Background and objectives

Hypertension is a major public health concern, contributing significantly to cardiovascular diseases, stroke, and chronic kidney disease. The burden of hypertension is increasing, particularly in low- and middle-income countries, due to lifestyle transitions, urbanization, and dietary changes. Kerala, despite its better health indicators, has a higher prevalence of hypertension compared to the national average, warranting targeted research and interventions. This study aims to determine the prevalence of hypertension among adults in Ernakulam district, Kerala, and to assess its association with sleep quality and other risk factors.

Materials and methods

A community-based cross-sectional study was conducted among 1,110 adults aged ≥30 years in Ernakulam district, Kerala, from September 2021 to March 2022. Cluster sampling using the probability proportional to size method was employed, selecting 30 clusters with 37 participants each. Data on sociodemographic characteristics, lifestyle factors, comorbidities, and sleep quality were collected using a structured questionnaire. Hypertension was defined per the Eighth Joint National Committee (JNC 8) guidelines based on blood pressure (BP) measurements obtained using a validated digital monitor. Sleep quality was assessed using the Pittsburgh Sleep Quality Index (PSQI), with a score of >5 indicating poor sleep quality. Consumption of caffeinated drinks was recorded based on self-reported intake of tea, coffee, soft drinks, or energy drinks, categorized into <2 cups, 2-4 cups, and >4 cups per day. Dyslipidemia, diabetes, and other comorbidities were self-reported based on prior medical diagnoses. Socioeconomic status (SES) was determined using the Kerala Public Distribution System’s ration card classification, with above poverty line (APL) and below poverty line (BPL) as categories. Data were analyzed using IBM SPSS Statistics for Windows, Version 21 (Released 2012; IBM Corp., Armonk, New York, United States), with multivariable logistic regression performed to determine associations between hypertension and risk factors. Statistical significance was set at p < 0.05.

Results

The mean age of participants was 54.46 ± 14.22 years. The prevalence of hypertension was 49.8% (95% CI: 49.34%-50.25%). Significant risk factors included poor sleep quality (aOR = 2.14, p < 0.001), consumption of caffeinated drinks (aOR = 11.43, p < 0.001), higher SES (aOR = 2.14, p < 0.001), and dyslipidemia (aOR = 12.804, p < 0.001).

Conclusion

Nearly half of the adult population in Ernakulam district has hypertension. Addressing modifiable risk factors such as poor sleep quality, caffeine consumption, higher SES, and dyslipidemia is crucial in hypertension prevention strategies.

## Introduction

Hypertension remains a significant global public health challenge, contributing substantially to cardiovascular diseases (CVD), stroke, and chronic kidney disease [1]. Recent estimates indicate that the global prevalence of hypertension exceeds 30%, with a rising trend in low- and middle-income countries [2]. In India, the prevalence was reported at 28.1% in 2023 [3], while in Kerala, it is notably higher at 43% [4]. This increasing burden, particularly in low- and middle-income countries, is driven by lifestyle transitions, dietary changes, and urbanization [5]. India has experienced a significant epidemiological shift, with noncommunicable diseases (NCDs) now surpassing communicable diseases as the leading cause of morbidity and mortality [6]. Despite Kerala’s superior health indicators compared to other Indian states, the high prevalence of hypertension remains a concern, potentially due to factors such as an aging population, excessive salt consumption, sedentary lifestyles, and genetic predisposition [7].

Often asymptomatic in its early stages, hypertension is commonly referred to as a "silent killer." If left uncontrolled, it substantially increases the risk of stroke, myocardial infarction, and chronic kidney disease [8]. Its development and progression result from a complex interplay of modifiable and nonmodifiable risk factors. Modifiable risk factors include an unhealthy diet, excessive salt intake, physical inactivity, obesity, smoking, and alcohol consumption. A high-sodium, low-potassium diet is strongly correlated with elevated blood pressure (BP), with studies demonstrating a clear link between excessive salt consumption and hypertension prevalence [9]. Similarly, obesity is a well-established risk factor, with evidence suggesting that overweight individuals have a two- to threefold higher risk of developing hypertension compared to those with normal weight [10]. Physical inactivity is another critical determinant, as sedentary lifestyles are associated with increased BP and heightened cardiovascular risk [11]. Additionally, tobacco use and excessive alcohol consumption contribute to endothelial dysfunction and arterial stiffness, further exacerbating hypertension risk [12].

Nonmodifiable risk factors include age, genetic predisposition, and family history of hypertension. Advancing age is a key determinant, with studies indicating that vascular stiffening and reduced arterial elasticity significantly increase hypertension risk [13]. Socioeconomic factors also play a role, as lower socioeconomic status (SES) is linked to higher stress levels, limited healthcare access, and unhealthy lifestyle behaviors [14].

Despite the growing burden of hypertension, awareness, treatment, and control rates remain suboptimal, underscoring the need for region-specific investigations to inform effective prevention and management strategies [2]. Given the increasing prevalence of hypertension and its associated risk factors, this study aims to determine the prevalence of hypertension among adults in Ernakulam district, Kerala. Additionally, it seeks to identify key modifiable and nonmodifiable risk factors to guide targeted interventions and public health policies. The findings of this study will contribute to a deeper understanding of the epidemiology of hypertension in the region and support the development of evidence-based prevention strategies.

## Materials and methods

A community-based cross-sectional study was conducted among 1,110 adults aged 30 years and above, residing in both rural and urban areas of Ernakulam district, Kerala, between September 2021 and March 2022. Ethical approval for the study was obtained from the Institutional Ethics Committee of Amrita Institute of Medical Sciences, Kochi, on January 19, 2021 (ECASM-AIMS-2021-012). A written informed consent in Malayalam was secured from all participants before data collection.

Individuals aged 30 years or older who had resided in Ernakulam district for at least six months were eligible for inclusion. However, individuals with known sleep disorders, intellectual disabilities, psychiatric illnesses, or those in a comatose state were excluded from the study.

The sample size was determined based on a hypertension prevalence of 43%, as reported in a 2018 study conducted in Ernakulam district [4]. Using the formula 4pq/d², where p = 43%, q = 100 - 43 = 57, and d = 10% of the prevalence, the estimated sample size was 530. Considering a design effect of two due to cluster sampling, the final sample size was increased to 1,060, with a total of 1,110 participants ultimately recruited. Cluster sampling using the probability proportional to size (PPS) method was employed, selecting 30 clusters, each consisting of 37 participants. One ward from the local body was randomly selected as a cluster, and participants were enrolled by visiting adjacent houses until the required number was achieved. All eligible adults within a household were included. This process was repeated across all 30 clusters to complete the sample.

BP was measured using an automatic digital BP monitor (OMRON HEM-8712) due to its reliability, ease of use, and feasibility in community-based studies. Before the study, the device was calibrated and validated against a standard mercury sphygmomanometer to ensure accuracy. Calibration was performed according to the manufacturer's guidelines, and periodic checks were conducted to maintain measurement reliability. Participants were seated in a quiet environment with their backs supported, feet flat on the floor, and legs uncrossed. Measurements were taken after a five-minute rest period, ensuring that participants had avoided caffeine, smoking, or physical activity for at least 30 minutes prior. Two readings were recorded at a 30-minute interval, and the average was considered. Hypertension was defined according to the Eighth Joint National Committee (JNC 8) criteria, classifying individuals as hypertensive if their systolic BP (SBP) was ≥ 140 mmHg, diastolic BP (DBP) was ≥ 90 mmHg, or if they were on antihypertensive medication [15].

Sleep quality was assessed as an independent risk factor for hypertension, given emerging evidence linking poor sleep to increased BP, autonomic dysfunction, and heightened cardiovascular risk. Sleep quality was assessed using the Pittsburgh Sleep Quality Index (PSQI), a validated instrument comprising 10 questions distributed across seven components: sleep duration, subjective sleep quality, sleep latency, habitual sleep efficiency, sleep disturbances, use of sleep medication, and daytime dysfunction. The total PSQI score ranges from 0 to 21, with higher scores indicating poorer sleep quality. A score exceeding 5 was considered indicative of poor sleep quality [16].

Sociodemographic variables, including age, sex, education, occupation, marital status, and area of residence, were recorded using a semi-structured questionnaire (questionnaire attached as a supplementary table in the Appendices). Anthropometric measurements were taken following standard protocols: weight was measured using a portable digital weighing scale, and height was measured using a constant-tension tape [17]. Body mass index (BMI) was calculated as weight (kg) divided by height (m²) and categorized according to the South Asian classification: underweight (<18.5 kg/m²), normal weight (18.5-22.9 kg/m²), overweight (23.0-24.9 kg/m²), and obesity (≥25.0 kg/m²) [18].

SES was assessed using the Kerala Public Distribution System's ration card classification. Individuals possessing blue or white ration cards were categorized as above poverty line (APL), while those with yellow or pink ration cards were classified as below poverty line (BPL). This classification was chosen as it serves as a practical and locally relevant socioeconomic indicator, widely used in public health research in Kerala. The ration card system is linked to income thresholds and access to subsidized food and healthcare, making it an appropriate measure for assessing socioeconomic disparities in health outcomes.

Lifestyle variables, including smoking status, alcohol consumption, caffeinated beverage intake, salt consumption, and physical activity levels, were assessed using a structured questionnaire. Participants were classified as current smokers (smoking at least one cigarette per day in the past 30 days), ex-smokers (quitting at least six months prior), and never-smokers. Alcohol consumption was categorized as current drinkers (consumed alcohol at least once in the past 30 days), former drinkers (abstained for at least six months), and never-drinkers.

Caffeinated beverage consumption is defined as the habitual intake of coffee, tea, or energy drinks at least once daily as self-reported by participants. It was categorized by type (tea, coffee, soft drinks, or combinations) and daily intake (<2 cups, 2-4 cups, >4 cups).

Daily salt intake was assessed through dietary recall and classified based on the WHO recommendations into <3 g/day, 3-5 g/day, and >5 g/day. Physical activity was categorized as sedentary (minimal activity), light (occasional walking/household chores), moderate (brisk walking, cycling, or gardening ≥150 minutes/week), and strenuous (intense exercise ≥75 minutes/week). All comorbidities, including dyslipidemia, diabetes, and cardiovascular diseases, were self-reported by participants.

Dyslipidemia was identified based on self-reported medical history, lipid profile results indicating abnormal levels of total cholesterol, low-density lipoprotein (LDL), high-density lipoprotein (HDL), or triglycerides, or a physician-diagnosed case of dyslipidemia, in accordance with standard diagnostic guidelines. However, specific lipid parameters such as LDL-c, HDL-c, triglycerides (TG), Lp (a), or non-HDL-c were not individually assessed or measured as part of this study. Similarly, diabetes and other chronic conditions were recorded based on prior medical diagnoses as reported by the participants.

Data were entered into MS Excel (Microsoft Corporation, Redmond, Washington, United States) and analyzed using IBM SPSS Statistics for Windows, Version 21 (Released 2012; IBM Corp., Armonk, New York, United States). Quantitative variables following a normal distribution were expressed as mean and standard deviation, while categorical variables were summarized as frequencies and percentages. Associations between hypertension and independent variables were assessed using the chi-square test. Variables with a p-value of <0.2 were considered for multivariable logistic regression analysis. Adjusted odds ratios (aORs) with 95% confidence intervals were calculated to determine associations between hypertension and risk factors, with statistical significance set at p < 0.05.

## Results

The mean age of the study population was 54.46 ± 14.22 years. A majority of the participants were females (n = 673, 60.6%), and there was a predominance of urban residents (n = 640, 57.7%). The overall prevalence of hypertension among adults aged 30 years and above in Ernakulam district was found to be 49.8% (95% CI: 49.34%-50.25%), indicating a substantial burden of the condition in the community. 

Approximately half of the participants (572, 51.5%) were aged ≤55 years, while 538 (48.5%)were older than 55 years. The study population was predominantly Hindu (539, 48.6%), followed by Christians (342, 30.8%) and Muslims (229, 20.6%). A significant proportion of participants lived in nuclear families (801, 72.2%), while 273 (24.6%) resided in joint families, and 36 (3.2%) in three-generation households.

The majority of participants 852 (76.8%) belonged to families with four or fewer members. Educational attainment was relatively low, with 697 (62.7%) having received less than 12 years of schooling. In terms of occupational status, homemakers constituted the largest group (466, 42%), followed by skilled laborers (246, 22.2%), unskilled laborers (158, 14.2%), and unemployed individuals (157, 14.1%). Only a small fraction (83, 7.5%) were professionals. The majority (1035, 93.2%) belonged to the APL category, whereas only 75 (6.8%) were classified as BPL.

The majority of the participants (1047, 94.3%) were nonsmokers. Among the smokers who accounted for only 41 (3.7%), the mean age of initiation of smoking was 20.78 ± 5.48 years. The majority of smokers (33, 80.4%) reported using cigarettes, while eight (19.5%) smoked beedis. Among current smokers, most (32, 78%) consumed between five and 10 units per day, while a smaller proportion (7, 17.1%) smoked fewer than five units per day, and two (4.9%) smoked more than 10 units per day.

Alcohol consumption was reported by 116 (10.5%) participants, while the majority (959, 86.3%) had never consumed alcohol. Among current drinkers (n = 115), brandy was the most commonly consumed alcoholic beverage (75, 65.2%), followed by whisky (27, 23.5%), rum (8, 6.9%), and toddy (5, 4.3%). A significant proportion of drinkers (54, 47%) reported consuming 10-20 drinks per month, whereas 24 (20.9%) consumed fewer than 10 drinks per month.

The majority of the participants (1059, 95.4%) consumed caffeinated drinks like tea, coffee, carbonated drinks, etc. The majority (875, 82.9%) reported drinking tea exclusively, while 132 (2.5%) consumed both tea and coffee. Most participants (992, 93.7%) consumed between two and four cups per day, whereas 34 (3.2%) reported consuming more than four cups daily. The average salt intake of the study participants was found to be 4.96 ± 0.68 g per day. About half of the participants (44.3%) had a sedentary lifestyle. The mean BMI of the participants was 24.53 ± 4.30. Less than a third (342, 30.8%) of the study participants had poor sleep quality as assessed by the PSQI questionnaire.

Physical activity levels indicated that nearly half of the participants (492, 44.3%) led a sedentary lifestyle, while 478 (43.1%) engaged in light physical activity. Only 133 (12%) performed moderate-intensity activities, and a mere seven (0.6%) engaged in strenuous physical activity. The mean BMI of the study population was 24.53 ± 4.30. Based on the BMI classifications, 463 (41.7%) of participants were categorized as obese, 242 (21.8%) were overweight, 322 (29%) had a normal BMI, and 83 (7.5%) were underweight. Sleep quality, as assessed using the PSQI, revealed that nearly one-third (342, 30.8%) of participants had poor sleep quality. 

After univariate analysis, the variables that had a p < 0.2 were taken into the multivariable model by the enter method. Poor sleep quality showed 2.32 times increased odds (95% CI: 1.7, 43.5) of being associated with hypertension compared to only 2.06 times in the unadjusted univariate analysis. As far as SES was concerned, people who were APL had 2.14 (95% CI: 1.16, 3.90) times more risk of having hypertension compared to people who are BPL after adjustment compared to 1.6 times in the unadjusted univariate analysis (Table 1).

**Table 1:** Sociodemographic characteristics, risk factors, and univariate analysis of hypertension APL: above poverty line; BPL: below poverty line; PSQI: Pittsburgh Sleep Quality Index

Variable	Total (N = 1110)	Hypertension yes (n = 553)	Hypertension no (n = 557)	Chi-square value	p-value
Age				0.133	0.716
≤55 years	572 (51.5%)	288 (50.3%)	284 (49.7%)		
>55 years	538 (48.5%)	265 (49.3%)	273 (50.7%)		
Sex				0.680	0.409
Male	437 (39.4%)	211 (48.3%)	226 (51.7%)		
Female	673 (60.6%)	342 (50.8%)	331 (49.2%)		
Religion				7.279	0.026
Hindu	539 (48.6%)	256 (47.5%)	283 (52.5%)		
Christian	342 (30.8%)	191 (55.8%)	151 (44.2%)		
Muslim	229 (20.6%)	106 (46.3%)	123 (53.7%)		
Type of family				15.399	<0.001
Nuclear	801 (72.2%)	398 (49.7%)	403 (50.3%)		
Joint	273 (24.6%)	148 (54.2%)	125 (45.8%)		
Three generation	36 (3.2%)	7 (19.4%)	29 (80.6%)		
Residence				4.864	0.027
Urban	640 (57.7%)	337 (52.7%)	303 (47.3%)		
Rural	470 (42.3%)	216 (46.0%)	254 (54.0%)		
Marital status				0.427	0.980
Ever married	1093 (98.4%)	544 (49.7%)	549 (50.3%)		
Never married	17 (1.6%)	9 (52.9%)	8 (47.1%)		
Education				8.328	0.215
<12 years of schooling	697 (62.7%)	356 (51.07%)	341 (48.9%)		
≥12 years of schooling	413 (37.3%)	197 (47.6%)	216 (52.4%)		
Occupation				2.091	0.719
Professional	83 (7.5%)	36 (43.4%)	47 (56.6%)		
Homemaker	466 (42.0%)	233 (50.0%)	233 (50.0%)		
Skilled labor	246 (22.2%)	127 (51.6%)	119 (48.4%)		
Unskilled labor	158 (14.2%)	76 (48.1%)	82 (51.9%)		
Unemployed	157 (14.1%)	81 (51.6%)	76 (48.4%)		
Socioeconomic status				4.002	0.045
APL	1035 (93.2%)	524 (50.6%)	511 (49.4%)		
BPL	75 (6.8%)	29 (38.7%)	46 (61.3%)		
Sleep quality (PSQI)				27.88	<0.001
Good	768 (69.2%)	342 (44.5%)	426 (55.5%)		
Poor	342 (30.8%)	211 (61.7%)	131 (38.3%)		
Smoking status				0.193	0.908
Nonsmoker	1047 (94.3%)	523 (50.0%)	524 (50.0%)		
Ex-smoker	22 (2.0%)	10 (45.5%)	12 (54.5%)		
Current smoker	41 (3.7%)	20 (48.8%)	21 (51.2%)		
Alcohol intake				14.086	<0.001
Never	959 (86.3%)	484 (50.5%)	474 (49.5%)		
Former drinker	36 (3.4%)	7 (19.4%)	29 (80.6%)		
Current drinker	115 (10.3%)	62 (53.4%)	54 (46.6%)		
BMI				169.88	<0.001
Underweight	83 (7.5%)	64 (77.1%)	19 (22.9%)		
Normal	322 (29.0%)	242 (75.2%)	80 (24.8%)		
Overweight	242 (21.8%)	79 (32.6%)	163 (67.4%)		
Obese	463 (41.7%)	168 (36.3%)	295 (63.7%)		
Dyslipidemia				64.08	<0.001
Yes	88 (8.0%)	88 (88.0%)	12 (12.0%)		
No	1022 (92.0%)	465 (46.0%)	545 (54.0%)		

**Figure 1:**
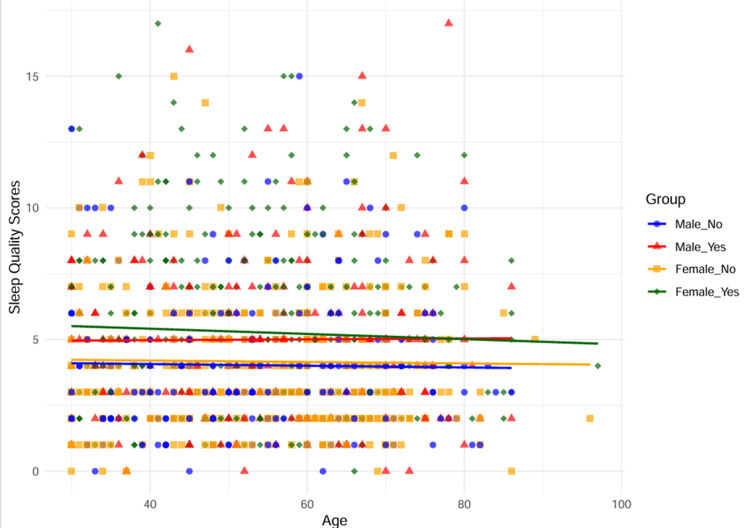
Scatter plot of age, sleep quality, gender, and hypertension Males without hypertension → blue circles; males with hypertension → red triangles; females without hypertension → orange squares; females with hypertension → green diamonds Each group is represented by a unique marker, with a regression line of the same color illustrating trends

The scatter plot illustrates the relationship between age and sleep quality scores across four groups based on sex and hypertension status: male without hypertension (blue circles), male with hypertension (red triangles), female without hypertension (orange squares), and female with hypertension (green diamonds). Each group has a corresponding regression line to depict the trend. The correlation coefficients (r-values) are close to zero for all groups, indicating a very weak or no linear relationship between age and sleep quality scores. Specifically, for males without hypertension (r = -0.0175, p = 0.7915) and males with hypertension (r = 0.0071, p = 0.9188), the negligible correlations suggest that age has no significant effect on sleep quality. Similarly, for females without hypertension (r = -0.0142, p = 0.7977) and females with hypertension (r = -0.0437, p = 0.4167), the associations remain weak and nonsignificant. Additionally, the 95% confidence intervals for all groups include zero, further confirming the lack of meaningful correlation (Figure 1).

The multivariable logistic regression analysis identified several significant risk factors for hypertension. Participants with poor sleep quality had 2.3 times higher odds of hypertension (aOR = 2.319, 95% CI: 1.704-3.156, p < 0.001) compared to those with good sleep quality. Caffeinated drink consumption was strongly associated with hypertension, with consumers having 11.4 times higher odds (aOR = 11.437, 95% CI: 5.181-25.247, p < 0.001) compared to nonconsumers. Individuals belonging to the APL category had significantly higher odds of hypertension (aOR = 2.135, 95% CI: 1.167-3.904, p = 0.014) than those from the BPL group.

Among lifestyle factors, former alcohol drinkers had significantly lower odds of hypertension (aOR = 0.137, 95% CI: 0.052-0.363, p < 0.001), indicating a possible protective effect of alcohol cessation. However, current alcohol consumption was not significantly associated with hypertension (aOR = 1.020, 95% CI: 0.641-1.621, p = 0.933).

In terms of BMI categories, overweight individuals had a lower risk of hypertension (aOR = 0.140, 95% CI: 0.098-0.198, p < 0.001), and underweight participants also exhibited a lower risk (aOR = 0.374, 95% CI: 0.177-0.792, p < 0.001) compared to obese individuals. This finding may indicate a potential obesity paradox, where some overweight individuals have better metabolic adaptation, or it may reflect survival bias, where chronic illnesses contribute to lower BMI. Dyslipidemia emerged as the strongest predictor of hypertension, with affected individuals having 12.8 times higher odds (aOR = 12.804, 95% CI: 6.498-25.228, p < 0.001) compared to those without dyslipidemia (Table 2).

**Table 2:** Multivariable logistic regression analysis of risk factors for hypertension Ref: reference category; APL: above poverty line; BPL: below poverty line

Variable	Unadjusted OR (95% CI)	p-value (OR)	Adjusted OR (95% CI)	p-value (aOR)
Sleep quality				
Poor sleep quality	2.006 (1.546-2.603)	<0.001	2.319 (1.704-3.156)	<0.001
Good sleep quality (ref)	Ref	—	Ref	—
Type of family				
Nuclear	4.091 (1.772-9.448)	0.002	2.014 (0.797-5.088)	0.139
Joint	4.905 (2.078-11.580)	0.001	2.068 (0.793-5.398)	0.138
Three generation (ref)	Ref	—	Ref	—
Area of residence				
Urban	1.308 (1.030-1.661)	0.028	1.175 (0.881-1.566)	0.272
Rural (ref)	Ref	—	Ref	—
Alcohol intake				
Ex-drinker	0.236 (0.103-0.545)	<0.001	0.137 (0.052-0.363)	<0.001
Current drinker	1.124 (0.764-1.654)	0.528	1.020 (0.641-1.621)	0.933
Nondrinker (ref)	Ref	—	Ref	—
Caffeinated drinks				
Yes	5.441 (2.637-11.229)	<0.001	11.437 (5.181-25.247)	<0.001
No (ref)	Ref	—	Ref	—
Socioeconomic status				
APL	1.627 (1.006-2.630)	0.047	2.135 (1.167-3.904)	0.014
BPL (ref)	Ref	—	Ref	—
BMI				
Normal	3.025 (1.849-4.950)	<0.001	1.046 (0.554-1.978)	0.890
Underweight	0.485 (0.294-0.800)	0.004	0.374 (0.177-0.792)	<0.001
Overweight	0.496 (0.309-0.797)	0.004	0.140 (0.098-0.198)	<0.001
Obese (ref)	Ref	—	Ref	—
Dyslipidemia				
Yes	8.595 (4.644-15.909)	<0.001	12.804 (6.498-25.228)	<0.001
No (ref)	Ref	—	Ref	—

## Discussion

The present study highlights a high prevalence of hypertension (49.8%) among adults above 30 years old in Ernakulam district. This finding is consistent with national and regional epidemiological studies, indicating an increasing burden of hypertension in India. Poor sleep quality, belonging to the APL category, consumption of caffeinated drinks, and dyslipidemia were identified as significant independent predictors of hypertension. Conversely, individuals who had quit alcohol exhibited a significantly reduced risk.

A slightly lower prevalence of hypertension (43%) was reported in a 2018 study conducted in Ernakulam district [4]. The overall absolute increase in prevalence was 27.6% (95% CI: 27.3, 27.9). According to a systematic review and meta-analysis, the prevalence of hypertension in India was estimated at 29.8% (95% CI: 26.7, 33.0), while a more recent study in 2022 reported an overall age-adjusted prevalence of 17.2% [19]. Urban areas showed a higher prevalence (18.3%) compared to rural populations (15.5%) [20]. Similarly, a 2023 study in the United States observed an urban-rural difference in hypertension prevalence, with participants in large rural areas having 17% higher odds of hypertension compared to urban counterparts, while those in small isolated rural areas exhibited a 19% higher risk [21].

The sociodemographic characteristics of the study population revealed a predominance of women (60.6%), aligning with findings from community-based health surveys, where women often constitute a larger proportion due to higher health-seeking behavior [22]. Regarding lifestyle factors, the majority of participants were nonsmokers (94.3%), with only 3.7% reporting current smoking. This figure is lower than the national average reported in the Global Adult Tobacco Survey (GATS) India (2016-2017), which estimated smoking prevalence at 11.2% among adults. The lower prevalence observed in this study may reflect regional tobacco control efforts and heightened awareness [23]. Alcohol consumption was reported by 10.5% of participants, a figure lower than the national average but comparable to other Kerala-based studies. Interestingly, former alcohol consumers exhibited a protective effect against hypertension. This finding that former alcohol drinkers had significantly lower odds of hypertension (aOR = 0.137, 95% CI: 0.052-0.363, p < 0.001) compared to nondrinkers suggests a potential protective effect of alcohol cessation. However, this association may be influenced by several factors. Individuals who quit alcohol may have also adopted healthier behaviors, such as improved diet and increased physical activity, which could contribute to a lower hypertension risk. Additionally, reverse causation is possible, as some individuals may have stopped drinking after being diagnosed with hypertension or other health conditions, leading to better BP control. Another explanation is the cardiovascular adaptation hypothesis, where moderate alcohol consumption prior to cessation may have had residual protective effects, whereas non-drinkers never had such exposure. Given these possibilities, the observed association requires cautious interpretation, and further longitudinal studies are needed to determine whether alcohol cessation itself lowers hypertension risk or if it is a marker for other beneficial lifestyle modifications. Similar findings were reported by Sesso et al., where heavy alcohol intake was associated with an increased risk of hypertension [24].

Notably, caffeine consumption was strongly associated with hypertension, with consumers facing an 11-fold higher risk compared to nonconsumers. A six-year follow-up study found that coffee consumers were more likely to experience persistent hypertension than nondrinkers (53.1% vs. 43.9%) [25]. Additionally, our study identified a significant association between SES and hypertension, with individuals belonging to the APL category facing higher risk. This may be attributed to increased consumption of processed foods and caffeinated beverages, which are known risk factors for hypertension. A cross-sectional study by Thrift et al. similarly found a significant association between higher SES and hypertension [26]. However, a cohort study conducted in South Asia in 2017 linked hypertension risk to factors such as age, low SES, current alcohol use, overweight, prehypertension, and dysglycemia [27]. The reversal of this trend in the present study suggests that urbanization and dietary transitions among higher-income groups may be contributing to elevated hypertension rates.

Poor sleep quality emerged as a significant predictor of hypertension, with individuals experiencing poor sleep having a 2.32-fold increased risk. This finding is consistent with studies by Gangwisch et al. [28], which emphasize the role of sleep disturbances in cardiovascular risk. Sleep deprivation has been linked to increased sympathetic activity, inflammation, and endothelial dysfunction, contributing to elevated BP. A study conducted in the Chinese population similarly found an association between PSQI scores and hypertension, with poor sleepers exhibiting greater odds of hypertension [29]. Among comorbid conditions, dyslipidemia demonstrated the strongest association with hypertension (OR: 12.8, 95% CI: 6.49-25.23), reinforcing the interplay between lipid metabolism disorders and hypertension. This aligns with studies by Otsuka et al., which highlight dyslipidemia as a major risk factor for hypertension and cardiovascular diseases [30].

Overall, our findings underscore the multifactorial nature of hypertension, with significant contributions from lifestyle factors, metabolic conditions, and SES. The observed association between higher SES and increased hypertension risk (OR: 2.14) supports existing literature suggesting that urbanization and dietary transitions in higher-income groups contribute to elevated hypertension rates. Public health interventions should, therefore, target both lifestyle modifications and metabolic risk factor management to mitigate the growing burden of hypertension in the population.

However, this study has certain limitations. The diagnosis of hypertension was based on single-day readings, which may not fully capture an individual's true BP status. Additionally, the PSQI assesses sleep quality over the past month, making it difficult to determine whether poor sleep quality was a recent development or a long-term condition. Similarly, caffeine consumption was self-reported, potentially leading to approximate estimates. Furthermore, recall bias may have influenced responses regarding the duration of hypertension, smoking, and alcohol consumption, as participants may have had difficulty accurately recalling past behaviors.

## Conclusions

Hypertension remains a significant public health concern in Ernakulam district, with multiple lifestyle and socioeconomic factors contributing to its high prevalence. This study underscores the complex interplay between behavioral habits, SES, and associated risk factors in shaping hypertension risk. Addressing these determinants requires a multifaceted approach that extends beyond clinical management to incorporate comprehensive public health strategies. Future efforts should prioritize targeted interventions that promote healthier lifestyle choices, such as balanced nutrition, physical activity, and sleep hygiene. Community-based initiatives for early detection and risk reduction, alongside policy-level actions to enhance awareness and accessibility of preventive healthcare, are crucial in mitigating the long-term burden of hypertension. Additionally, longitudinal studies are needed to establish causal pathways and assess the effectiveness of sustained behavioral interventions. By integrating clinical, behavioral, and policy-driven strategies, a more holistic approach to hypertension prevention and control can be achieved, ultimately reducing morbidity and improving population health outcomes.

## Appendices

**Table 3:** Sociodemographic questionnaire Credits: Aparna Ajay, Paul T Francis, Aswathy S, Rajeesh R Nair, CV Lalithambika

Prevalence and risk factors of hypertension among adults in Ernakulam district, Kerala: a cross-sectional study		
Section	Question	Response options
General information	Date	
	Cluster number	
	Cluster name	
	Case number	
	Case number in cluster	
	Address	
	Phone number	
	Name	
	Age	
	Sex	Male/female/transgender
Sociodemographic profile	Religion	1) Hindu 2) Christian 3) Muslim 4) Others
	Type of family	1) Nuclear 2) Joint 3) Three-generation
	Area of residence	1) Urban 2) Rural
	Marital status	1) Unmarried 2) Married 3) Legally divorced 4) Living in separation 5) Widow
	Education	1) Illiterate 2) Primary (1-4) 3) Middle (5-7) 4) High school (8-10) 5) Higher secondary (11-12) 6) Graduation 7) Postgraduation
	Occupation	1) Professional 2) Homemaker 3) Skilled 4) Unskilled 5) Unemployed
	Total number of family members	
	Family income (earning members)	
	Per capita income	
	Ration card color	1) White 2) Blue 3) Pink 4) Yellow 5) No ration card
Lifestyle factors	Smoking history	1) Nonsmoker 2) Ex-smoker 3) Current smoker
	Age of smoking initiation	
	Type of tobacco	
	Number of units smoked per day	
	If ex-smoker, stopped since	
	Alcohol intake	1) Never 2) Former drinker 3) Current drinker
	Type of alcohol consumed	
	If a former drinker, stopped since	
	Drinking frequency per month	
	Number of drinks per day	
	Caffeinated drink consumption	Yes/no
	If yes, type	1) Tea 2) Coffee 3) Soft Drinks 4) Energy drinks 5) Others:
	Cups per day	
	Average salt intake per day	
	Physical activity level	1) Sedentary 2) Light 3) Moderate 4) Strenuous
Health history	Current health problems	No/yes

Disease	Duration (years)	Treatment (yes/no)	Family History (yes/no)
Hypertension			
Diabetes			
Cardiovascular diseases			
Stroke			
Chronic respiratory disease			
Malignancy			
Arthritis			
Renal problems			
Depression			
Others (specify)			

Anthropometric and blood pressure measurements
Height (cm)
Weight (kg)
BP measurements
Reading 1
Reading 2 (after 30 mins)
